# Yifei Xuanfei Jiangzhuo formula, a Chinese herbal decoction, improves memory impairment through inhibiting apoptosis and enhancing PKA/CREB signal transduction in rats with cerebral ischemia/reperfusion

**DOI:** 10.3892/mmr.2015.3962

**Published:** 2015-06-18

**Authors:** LIN WU, QING-SHAN ZHAO, TIAN-WEI LI, HAI-YUAN LI, QING-BI WANG, XIN-YA BI, XIN-KUN CAI, NONG TANG

**Affiliations:** 1 Guangxi Scientific Experimental Center of Traditional Chinese Medicine, Guangxi University of Chinese Medicine, Nanning, Guangxi 530001, P.R. China; 2 Guangxi Key Laboratory of Chinese Medicine Foundation Research, Guangxi University of Chinese Medicine, Nanning, Guangxi 530001, P.R. China; 3 Graduate School, Hunan University of Chinese Medicine, Changsha, Hunan 410208, P.R. China; 4 Department of Internal Neurology, The First Affiliated Hospital, Guangxi University of Chinese Medicine, Nanning, Guangxi 530023, P.R. China

**Keywords:** Chinese medicine, vascular dementia, memory impairment, ischemia/reperfusion, apoptosis, signal transduction

## Abstract

Apoptosis and the dysfunction of the cyclic adenosine monophosphate (cAMP)/protein kinase A (PKA)/cAMP-responsive element binding protein (CREB) signaling pathway have a key role in memory impairment in vascular dementia (VaD), a challenging clinical problem. Yifei Xuanfei Jiangzhuo formula (YXJF), a Chinese herbal decoction, has been used to treat VaD in clinical practice and has produced positive outcomes; however, convincing evidence is currently lacking. The present study aimed to investigate the effects of YXJF on memory impairment in rats with cerebral ischemia/reperfusion and to explore the underlying mechanism. YXJF ameliorated memory impairment in rats with cerebral ischemia/reperfusion, inhibited hippocampal apoptosis in a dose-dependent manner and attenuated increases in the protein expression of B-cell lymphoma 2 (Bcl-2)-associated X protein as well as c-Jun and a reduction in Bcl-2 protein expression in the hippocampal tissue of the rats. Furthermore, administration of YXJF significantly increased the protein expression of PKA C-α and CREB, and promoted CREB phosphorylation. The results indicated that YXJF improves memory impairment through inhibiting apoptosis and enhancing PKA/CREB signal transduction in rats with cerebral ischemia/reperfusion.

## Introduction

Vascular dementia (VaD) is the second most common type of dementia after Alzheimer's disease (AD) among the elderly, accounting for approximately a quarter of all cases of dementia in developed countries (1). The disease is characterized by a progressive impairment of memory, which severally interferes with the life quality of the patients and their families when the loss develops to a certain degree (2). At present, VaD has become a major public problem and a challenging clinical one (3) with a marked increase among the elderly population in spite of limited success in the improvement of memory impairment.

VaD is considered to be caused by a variety of cerebral vascular diseases, including cerebral infarction and cerebral hemorrhage due to vascular lesions which restrict blood supply and damage the brain regions being important for memory, cognition and behavior (4,5). Although the exact pathogenesis has not yet been fully elucidated, it is now apparent that cerebral hypoperfusion is a main underlying mechanism for VaD. Ischemia/reperfusion leads to energy metabolic dysfunction and oxidant stress, thus triggering cell apoptosis and subsequent impairment of the brain, including the hippocampus, which has a key role in the regulation of memory function (6,7). Apoptosis is an active process of cell death which occurs in numerous important physiological and pathophysiological conditions. Following ischemia/reperfusion, the expression of apoptosis-associated proteins, including B-cell lymphoma 2 (Bcl-2), Bcl-2-associated X protein (Bax) and P53, are altered, which triggers and deteriorates apoptosis (8,9). Therefore, inhibiting apoptosis is a feasible strategy for the treatment of VaD.

Furthermore, accumulating evidence indicated that the cyclic adenosine monophosphate (cAMP)/protein kinase A (PKA)/cAMP-responsive element binding protein (CREB) signal transduction system has a close association with learning and memory function in mammals (10–12). cAMP is a ubiquitous secondary messenger that is strongly associated with hippocampal synaptic plasticity and memory (13). Elevated cytoplasmic cAMP promotes the activity of PKA, and PKA subsequently phosphorylates and activates CREB (12,14). Activated CREB consequently binds to the cAMP response element (CRE) of target genes, thus regulating memory, particularly long-term memory formation (10). Injection of cAMP into the lateral ventricle was shown to ameliorate experimental amnesia in mice (13), while PKA inhibitors impaired long-term memory formation in day-old chicks (14). Moreover, intra-hippocampal infusion of CREB antisense oligonucleotides significantly impaired long-term memory in rats (15). In rats with microsphere embolism-induced cerebral ischemia and memory defects, the activity of cAMP/PKA/CREB was significantly decreased in the hippocampus, as indicated by decreased cAMP content, PKA levels and CREB phosphorylation, while enhancement of the activity of this signaling pathway improved the memory function in rats (16,17).

In China, herbal medicines have been widely used to treat dementia for hundreds of years, and are believed to achieve beneficial outcomes; however, scientific evidence for their potency is currently lacking. Recently, Yifei Xuanfei Jiangzhuo formula (YXJF), a Chinese herbal decoction composed of nine Chinese herbs based on the differentiation of symptoms embodying the theory of Traditional Chinese Medicine, is used to treat VaD. A previous study by our group reported that the memory as well as life quality of patients with VaD were significantly improved after YXJF treatment (18), suggesting that YXJF is likely to be suitable for VaD treatment. However, the underlying mechanisms have yet to be fully elucidated. The present study aimed to investigate the effects of YXJF on memory impairment in a rat model of cerebral ischemia/reperfusion-induced VaD and to explore the underlying mechanisms, including anti-apoptotic effects and enhancement of cAMP/PKA/CREB signal transduction, which provided convincing evidence for the clinical efficacy of YXJF.

## Materials and methods

### Materials and reagents

Huperzine A was purchased from Shanghai Fudan Forward Pharmaceutical Co., Ltd. (Shanghai, China). Piracetam was obtained from Northeast General Pharmaceutical Factory (Shenyang, China). The *In situ* Cell Death Detection kit, peroxidase was from Roche (Basel, Switzerland). Protein lysis buffer for western blot was provided by Solarbio Science & Technology Co., Ltd. (Beijing, China). Antibodies directed to Bax (rabbit anti-rat; cat. no. 2772S; 1:800), c-Jun (mouse anti-rat; cat. no. 2315S; 1:500), Bcl-2 (rabbit anti-rat; cat. no. 2876S; 1:1,000), PKA C-α (rabbit anti-rat; cat. no. 4782S; 1:600), CREB (rabbit anti-rat; cat. no. 4820; 1:800), and phosphorylated CREB (p-CREB; rabbit anti-rat; cat. no. 4276; 1:500) were all from Cell Signaling Technology (Danvers, MA, USA). An antibody against β-actin (mouse anti-rat; cat. no. TA-09; 1:1,000) and secondary antibodies against peroxidase-conjugated goat anti-mouse IgG (cat. no. ZB-2305; 1:3,000) and peroxidase-conjugated goat anti-rabbit IgG (cat. no. ZB-2301; 1:3,000) were obtained from Zhongshan Jinqiao Biotechnology Co., Ltd. (Beijing, China). Notoginsenoside R1, astragaloside IV, amygdalin, platycodin D and ginsenoside Rg1 were purchased from Chengdu Must Biotechnology Co., Ltd. (Chengdu, China). Ginsenoside Rb1 was from National Institutes for Food and Drug Control (Beijing, China).

### Preparation of the decoction

**Figure 1:**
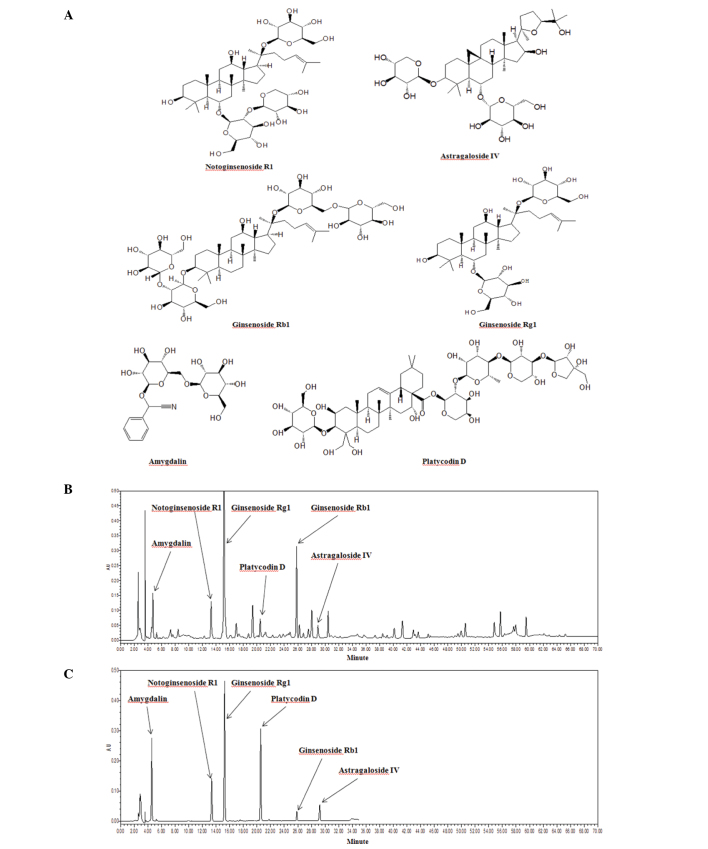
(A) Chemical structures of notoginsenoside R1, astragaloside IV, ginsenoside Rg1, ginsenoside Rb1, amygdalin and platycoldin D. HPLC chromatogram of (B) Yifei Xuanfei Jiangzhuo formula and (C) the reference compounds. The information for each peak is indicated. HPLC, high-performance liquid chromatography.

YXJF, the Chinese herbal decoction, was produced by extraction of the following nine Chinese herbs: The tuberous root of *Platycodon gradiflorus* (Jacq.) A. DC. (*Campanulaceae*), the seed of *Prunus armeniacae* L. (*Rosaceae*), the rhizome of *Panax ginseng* C. A. Mey. (*Araliaceae*), the tuberous root of *Ophiopogon japonicas* (L. f) Ker-Gawl. (*Liliaceae*), the root of *Astragalus membranaceus* (Fisch.) Bge. var. mongholicus (Bge.) Hsiao (*Leguminosae*), the rhizome of *Panax notoginseng* (Burk.) F. H. Chen (*Araliaceae*), the dried fruit of *Perilla frutescens* (L.) Britt. (*Labiatae*), the rhizome of *Acorus tatarinowii* Schott (*Araceae*), and the rhizome (stir-fried with wine) of *Rheum palmatum* L. (*Polygonaceae*). The herbs were purchased from Jiangyin Tianjiang Pharmaceutical Co., Ltd. (Jiangyin, China), and the voucher specimens were deposited at the First Affiliated Hospital, Guangxi University of Chinese Medicine (Nanning, China). All herbs were authenticated by Professor Lin Wu. The processed herbs were normatively prepared following the Good Manufacturing Practice specific to Chinese Herbal Medicine (19). In the decoction using water as a solvent, the percent ratios based on weight of the nine herbs were 8.62, 8.62, 12.93, 12.93, 12.93, 12.93, 12.93, 12.93 and 5.18, respectively. The concentration of the herbs in the formula was 3.39 g/ml, and the decoction was kept in the fridge at 4°C. The decoction was warmed in water at 37°C for 10 min prior to its administration to the rats. High performance liquid chromatography (HPLC; Milford, MA, USA), including Binary HPLC Pump 1525, a Photodiode Array Detector 2998 and a SunFire™ C_18_ column (4.6 mm × 250 mm; 5 *µ*m) was employed to analyze the chemical profile of the decoction. The mobile phase was pumped at a flow-rate of 1 ml/min and a 20 *µ*l sample was injected into the system. The chemical structure of each component and the HPLC chromatogram are shown in Fig. 1. Comparison with the reference compounds showed that the decoction contained notoginsenoside R1, astragaloside IV, ginsenoside Rg1, ginsenoside Rb1, amygdalin, platycodin D and other unidentified components.

### VaD rat model and treatments

Male Sprague-Dawley rats (n=210; age, 8-weeks old; weight, 200–230 g) were purchased from Hunan Slaccas Jingda Laboratory Animal Co., Ltd. (Changsha, China). The rats were housed in a room with a 12-h light/dark cycle and temperature- and humidity-control (24±2°C and 65±5%). All rats had free access to food and water throughout the experiments. After 1 week of acclimatization, the rats were subjected to the Morris water maze test to ensure their normal memory. Then, a rat model of VaD was induced by repeated occlusion of the common carotid artery followed by reperfusion, as previously described, with certain modifications (20,21). Briefly, the animals were randomly divided into sham-operated and operated groups. The rats were firstly anesthetized with chloral hydrate (300 mg/kg, i.p.; Beyotime Institute of Biotechnology Co., Ltd., Shanghai, China), and then bilateral common carotid arteries of rats in the operated group were ligated with 0 type surgical silk through a midline neck incision, and 0.3 ml blood was obtained from the tail vein. After 20 min, bilateral common carotid arteries were opened for 10 min by loosening the silk. This course was repeated three times, which resulted in cerebral ischemia/reperfusion in the rats. The rats in the sham-operated group received the same surgical operation without occlusion of the common carotid arteries. During the surgical procedures, the carotid sheath and the vagus nerve of rats were preserved from damage.

After 10 days of operation, all animals performed the Morris water maze test. The rats in the operated group (OPER; n=120) were randomly assigned to six sub-groups based on various treatments: The model group (MOD; n=20) receiving an equal volume of distilled water by oral gavage, the low-dose YXJF group (LDY; n=20) receiving low-dose YXJF (6.09 g/kg/day), the medium-dose YXJF group (MDY; n=20) receiving a medium dose YXJF (12.18 g/kg/d), the high-dose YXJF group (HDY; n=20) receiving high-dose YXJF (24.36 g/kg/day), the huperzine A group (HUA; n=20) receiving huperzine A (0.15 mg/kg/day) and the piracetam group (PIR; n=20) receiving piracetam (0.20 g/kg/day). The rats in the sham-operated group (SHAM; n=22) were, as the control, given an equal volume of distilled water. After 30 days of treatment, the rat memory was evaluated using the Morris water maze test.

The experiments were approved by the Ethics Committee of Guangxi University of Chinese Medicine (Nanning, China). All procedures were conducted in accordance with the internationally accepted Principles for Laboratory Animal use and Care and the Chinese Animal Welfare Legislation.

### Morris water maze test

Spatial learning and memory was evaluated by the Morris water maze test according to a previously described procedure with certain modifications (22). The maze consisted of a grey circular tank of 120 cm in diameter and 50 cm in height. The tank was filled with water kept at 22±1°C and divided into four quadrants. A circular platform of 10 cm in diameter was in the center of the second quadrant and submerged ~2.0 cm below the water surface. To ensure the platform was invisible to the rats, the water was made opaque using a non-toxic water-soluble black dye (carbon black ink; 1 ml/56 l; Guilin Fuxuan Stationary Co., Ltd., Guilin, China). All rats were released into the water (facing the sidewall) to search for the hidden platform at four distinct starting quadrant points, respectively, and each rat was given four trials a day (one trial for each quadrant). The time that rats took to find the submerged platform was recorded as the escape latency. Each rat was allowed to reach the platform within 60 sec and remain on it for 10 sec. If the rat did not find the platform within 60 sec, it was manually directed toward the platform and placed on the platform for 10 sec, and the escape latency was recorded as 60 sec. The rats were trained over five consecutive days. On day six, the platform was removed, and it was determined whether the rats found the expected location of the platform in a memory-dependent manner. The frequency at which the rat passed the targeted point where the platform had been located within 60 sec was recorded. The performance of the rats was recorded using a video camera and analyzed using WMT-100 Morris software (Chengdu Taimeng Software Co., Ltd., Chengdu, China).

### Transmission electron microscopic observation

After treatment and subsequent memory evaluation, the rats were randomly selected to be anesthetized with chloral hydrate (300 mg/kg, i.p.) and then perfused transcardially with normal saline followed by ~200 ml 2% paraformaldehyde and 2.5% glutaraldehyde (Tianjin Yongda Chemical Reagent Development Centre, Tianjin, China) to pre-fix the brain tissue. The brains were removed and fixed in 3% glutaraldehyde. The neuron ultrastructure in the hippocampal CA1 region was observed by transmission electron microscopy (TEM) according to a previously described method with certain modifications (23). In brief, the hippocampal CA1 area of the rats was rapidly excised, and then cut into pieces of 1 mm × 1 mm × 2 mm. The pieces were fixed in 3% glutaraldehyde for 2 h and post-fixed in 1% osmic acid (Beijing Zhongjingkeyi Technology Co., Ltd., Beijing, China) for 1.5 h. After that, the pieces were de-hydration-fixed by conventional ethanol and acetone treatments, embedded in ethoxyline resin 618 (Beijing Zhongjingkeyi Technology Co., Ltd.) and sliced using an ultra-thin slicer (UC7; Leica Microsystems, Wetzlar, Germany). Finally, the slices were double-stained with uranyl acetate (Beijing Zhongjingkeyi Technology Co., Ltd.) and lead citrate (Beijing Zhongjingkeyi Technology Co., Ltd.), and observed and filmed under a Hitachi-7650 TEM (Hitachi, Tokyo, Japan).

### Apoptosis analysis by terminal deoxynucleotidyl transferase-mediated (dUTP) nick end labeling (TUNEL) staining

Apoptosis was analyzed using TUNEL staining on paraffin-embedded sections according to a previously described procedure with certain modifications (24). Briefly, the rats were randomly selected to be anesthetized with chloral hydrate, and then perfused transcardially with normal saline followed by ~200 ml 4% paraformaldehyde (Tianjin Yongda Chemical Reagent Development Centre) to pre-fix the brain tissue. The brains were excised and fixed in 4% paraformaldehyde, embedded in paraffin and then cut into a series of sections. After de-paraffinization and re-hydration, the sections were rinsed in 0.1 M phosphate buffered saline (PBS) twice and incubated in Proteinase K working solution (10 *µ*g/ml in 10 mM Tris/HCl, pH 7.4–8.0) for 15 min at 37°C. After being rinsed in PBS again, the sections were treated with fluorescein (green)-labeled dUTP solution containing 10% TdT. A negative control was produced using dUTP, while a positive control was produced using DNase 1. The hippocampal CA1 region was observed and photographed at a magnification of ×200 using a fluorescence microscope (Olympus BX-60; Olympus, Tokyo, Japan), with green fluorescence indicating TUNEL-positive cells. After imaging, converter-POD was added on the slides. The sections were then counterstained with diaminobenzidine and hematoxylin (Sinopharm Chemical Reagent Co., Ltd., Shanghai China). Apoptotic cells represented by TUNEL-positive cells with fluorescent granules in the nuclei were observed using a microscope (BX-60; Olympus, Tokyo, Japan). At a magnification of ×400, five microscopic fields were randomly selected to count the TUNEL-positive cells.

### Western blot analysis

Protein expression in hippocampal tissue was analyzed by western blotting (25). The hippocampal homogenates were prepared using protein lysis buffer for western blot, and tissue debris was subsequently removed by centrifugation (12,000 × g for 10 min). The protein concentration of each sample was analyzed. After addition of sample buffer, the protein was heated for 10 min at 95°C and then separated by SDS-PAGE (Sigma-Aldrich, St. Louis, MO, USA). The protein was transferred to a polyvinylidene difluoride membrane (EMD Millipore, Billerica, MA, USA). The membrane was incubated with the appropriate primary antibody overnight at 4°C and then with the horseradish peroxidase-conjugated secondary antibody for 1.5 h at room temperature. The targeted proteins were detected by enhanced chemiluminescence (Amersham, Piscataway, NJ, USA) and images were obtained and analyzed using UVP GDS-8000 system (Thermo Fisher Scientific, Rockford, IL, USA).

### Statistical analysis

Values are expressed as the mean ± standard error. When multiple comparisons were performed, the significance was determined using one-way analysis of variance. Student's t-test was used to analyze differences between two groups. P<0.05 was considered to indicate a statistically significant difference. All data were analyzed with SPSS 16.0 for Windows (SPSS, Inc., Chicago, IL, USA).

## Results

### YXJF improves memory impairment in rats with cerebral ischemia/reperfusion

**Figure 2:**
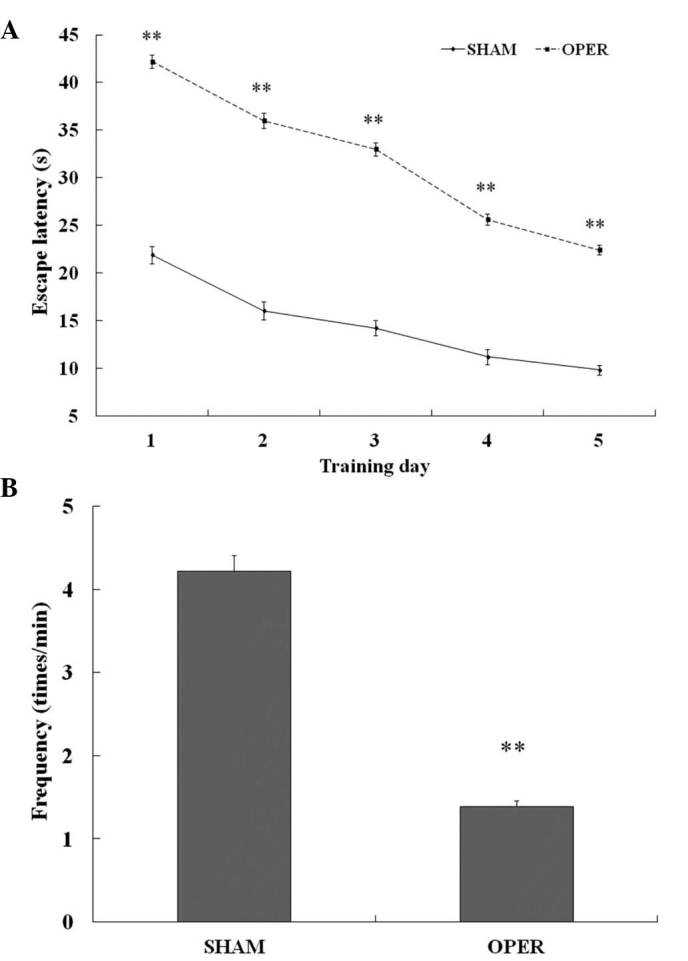
Morris water maze test in the sham-operated and the operated groups prior to treatment. After 10 days of rats receiving operation or sham operation, memory function was evaluated by the Morris water maze test. (A) Changes in escape latency of rats in the operated group (n=120) and the sham-operated group (n=22). (B) Frequency of the rats passing the potential platform within 60 sec. Values are expressed as the mean ± standard error. ^**^P<0.01 vs. sham group. OPER, operated group.

In the present study, a rat model of VaD was established by cerebral ischemia reperfusion. After operation (prior to treatment), the escape latency of the rats in the operated group was significantly longer than that in the sham-operated group (all P<0.01; Fig. 2A) within five consecutive training days, and the frequency at which the rats passed the potential platform was significantly decreased compared to that in the sham-operated group (P<0.01; Fig. 2B). The results indicated that the memory function of rats was severely impaired by cerebral ischemia/reperfusion, suggesting that the animal model of VaD was successfully induced.

**Figure 3:**
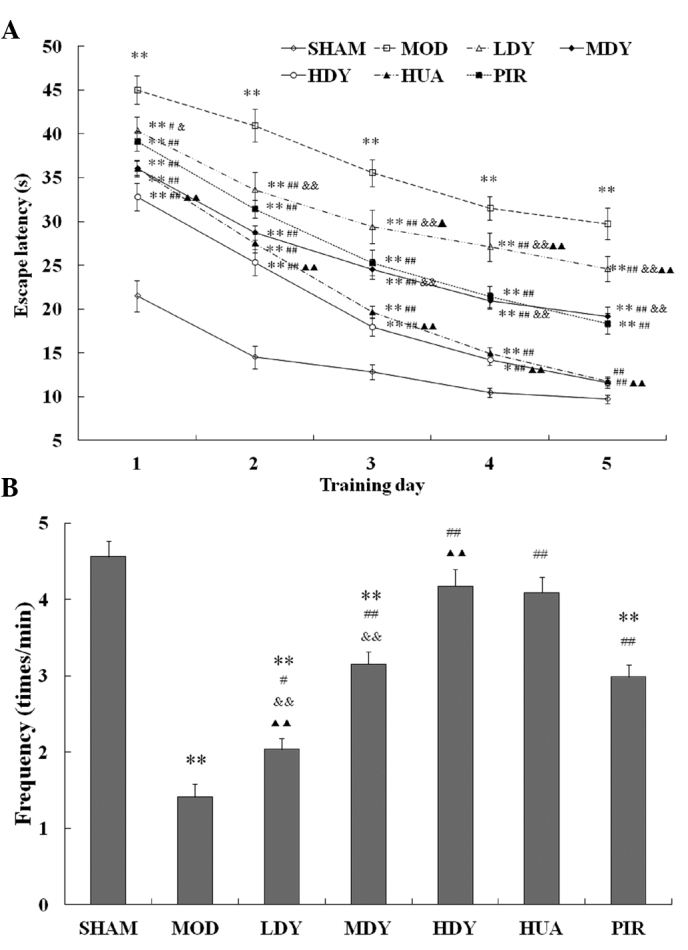
Effects of YXJF on the memory function of rats with cerebral ischemia/reperfusion. After treatment for 30 days, memory function of rats was evaluated by the Morris water maze test. (A) Escape latency. (B) Frequency of the rats passing the potential platform within 60 sec. Values are expressed as the mean ± standard error (n=20-22). ^*^P<0.05, ^**^P<0.01 vs. SHAM; ^#^P<0.05, ##P<0.01 vs. MOD; ^&^P<0.05, ^&&^P<0.01 vs. HUA; ^▲^P<0.05, ^▲▲^P<0.01 vs. PIR. MOD, model group; LDY, low-dose YXJF group; MDY, medium-dose YXJF group; HDY, high-dose YXJF group; HUA, huperzine A group; PIR, piracetam group; YXJF, Yifei Xuanfei Jiangzhuo formula.

Administration of YXJF markedly shortened the escape latency in a dose-dependent manner when compared to that in the model group (P<0.05 or P<0.01; Fig. 3A) within five consecutive trial days, and remarkably increased the frequency at which the rats passed the hypothetical platform within 60 sec (P<0.05 or P<0.01; Fig. 3B). In addition, the escape latency and frequency in the high-dose YXJF group were similar to those in the huperzine A group (P>0.05), while the escape latency was shorter and the frequency was higher compared with that in the piracetam group (all P<0.01). There was no difference in escape latency or frequency between the medium-dose YXJF group and the piracetam group (all P>0.05). These results demonstrated that YXJF improved cerebral ischemia/reperfusion-induced memory impairment in rats, providing evidence for the potential anti-dementia action of YXJF.

### YXJF inhibits apoptosis in the hippocampal CA1 region

**Figure 4:**
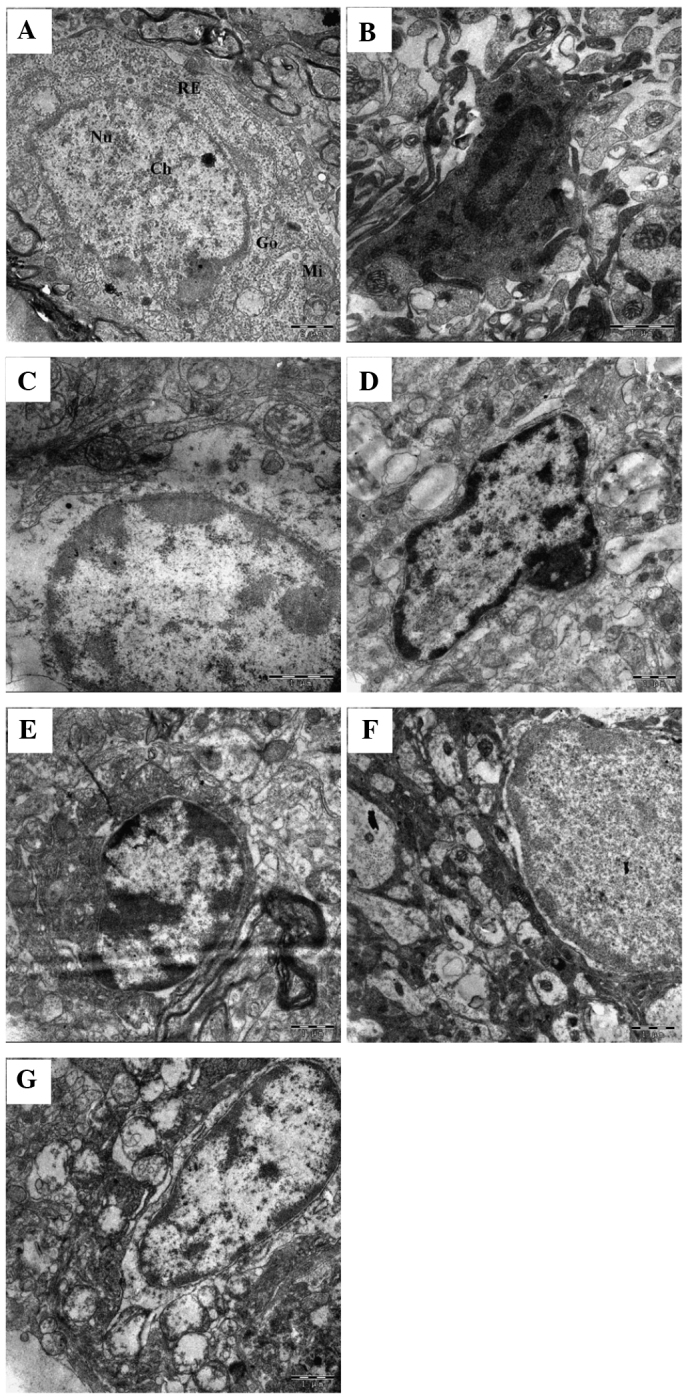
Effect of YXJF on neuron ultrastructure in hippocampus CA1 region. After treatment and subsequent memory evaluation, neuron ultra-structure in hippocampus CA1 region was examined by transmission electron microscopy. (A) Sham-operated group (scale bar, 2 *µ*m); (B) model group; (C) low-dose YXJF group; (D) medium-dose YXJF group; (E) high-dose YXJF group; (F) huperzine A group; (G) piracetam group (scale bar, 1 *µ*m). Nu, nucleus; Ch, chromosome; Go, golgi body; Mi, mitochondria; RE, rough endoplasmic reticulum; YXJF, Yifei Xuanfei Jiangzhuo formula.

As shown in Fig. 4, the neurons in the hippocampal CA1 region in the model group (Fig. 4B) exhibited typical ultrastructural characteristics of apoptosis, including smaller cells with nuclear condensation, chromatin margination, unchanged mitochondria and increased blebbing compared to that in the sham-operated group (Fig. 4A). After YXJF treatment (Fig. 4C–E), the neuronal ultrastructure was significantly improved, indicating a reduction of apoptosis. In addition, huperzine A (Fig. 4F) and piracetam (Fig. 4G) also ameliorated the apoptosis-associated ultrastructural changes in the neurons.

**Figure 5:**
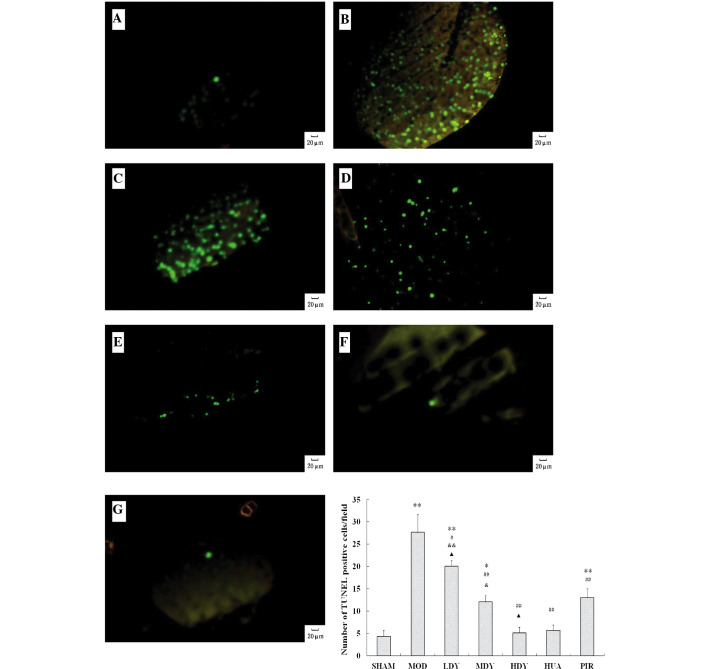
Apoptosis analysis by TUNEL staining. After treatment and subsequent memory evaluation, apoptosis in hippocampus CA1 region was analyzed by TUNEL staining. Green fluorescence indicates TUNEL-positive cells in each group. (A) SHAM group; (B) MOD group; (C) LDY group; (D) MDY group; (E) HDY group; (F) HUA group; and (G) PIR group (magnification, ×200). (H) Number of TUNEL-positive cells per field counted at a magnification of ×400 in each group. Values are expressed as the mean ± standard error. ^*^P<0.05, ^**^P<0.01 vs. SHAM; ^#^P<0.05, ^##^P<0.01 vs. MOD; ^&^P<0.05, ^&&^P<0.01 vs. HUA; ^▲^P<0.05 vs. PIR. MOD, model group; LDY, low-dose YXJF group; MDY, medium-dose YXJF group; HDY, high-dose YXJF group; HUA, huperzine A group; PIR, piracetam group; YXJF, Yifei Xuanfei Jiangzhuo formula.

In order to further confirm the anti-apoptotic action of YXJF, apoptosis in the hippocampal CA1 region was analyzed by TUNEL staining. Fig. 5 shows that TUNEL-positive cells in the model group (Fig. 5B and H) were significantly increased compared to those in the sham-operated group (P<0.01; Fig. 5A and H), suggesting that cerebral ischemia/reperfusion led to apoptosis in the hippocampus. In the treatment groups, YXJF inhibited apoptosis in a dose-dependent manner (Fig. 5C–E and H), as indicated by fewer TUNEL-positive cells compared with those in the model group (P<0.05 or P<0.01). In addition, huperzine A (Fig. 5F and H) and piracetam (Fig. 5G and H) also reduced apoptosis with no difference in TUNEL-positive cells between the high-dose YXJF and the huperzine A groups, as well as between the medium-dose YXJF and the piracetam groups (Fig. 5H). These results indicated that YXJF inhibited apoptosis in the hippocampal CA1 region of rats with cerebral ischemia/reperfusion.

### Effects of YXJF on the expression of apoptosis-associated proteins in the hippocampus

**Figure 6:**
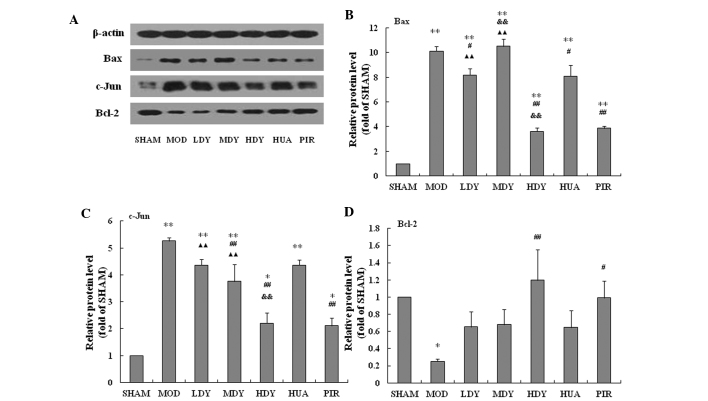
Protein expression of Bax, Bcl-2 and c-Jun in the hippocampus. (A) After treatment and subsequent memory evaluation, the hippocampus was harvested and the protein expression was assessed by western blot analysis. (B-D) Quantified protein levels of (B) Bax (C) c-Jun and (D) Bcl-2 normalized to β-actin. Values are expressed as the mean ± standard error.^*^P<0.05, ^**^P<0.01 vs. SHAM; ^#^P<0.05, ^##^P<0.01 vs. MOD; ^&&^P<0.01 vs. HUA; ^▲^P<0.05, ^▲▲^P<0.01 vs. PIR. Bcl-2, B-cell lymphoma 2; Bax, Bcl-2-associated X protein; MOD, model group; LDY, low-dose YXJF group; MDY, medium-dose YXJF group; HDY, high-dose YXJF group; HUA, huperzine A group; PIR, piracetam group; YXJF, Yifei Xuanfei Jiangzhuo formula.

To explore the underlying mechanisms of the inhibition of apoptosis by YXJF, the protein expression of Bax, Bcl-2,and c-Jun in the hippocampus was analyzed (Fig. 6). Compared with that in the sham-operated group, the protein expression of Bax and c-Jun in the model group was significantly increased, while that of Bcl-2 was markedly reduced, suggesting that cerebral ischemia/reperfusion-induced apoptosis was mediated by changes in the protein expression of Bax, Bcl-2 and c-Jun in the hippocampus. Administration of YXJF obviously decreased the protein expression of Bax and c-Jun, while enhancing Bcl-2 protein expression. Huperzine A significantly reduced Bax, but decreased c-Jun and increased Bcl-2 protein expression only to a certain extent, while piracetam markedly restrained Bax and c-Jun, and promoted Bcl-2 protein expression. Treatment with high-dose YXJF and piracetam had similar effects on the protein expression of the three apoptotic proteins. These results supported that YXJF inhibits apoptosis through reducing the protein expression of Bax and c-Jun, and promoting Bcl-2 protein expression, and its mechanism of action appears to be more similar to that of piracetam than that of huperzine A.

### Effects of YXJF on the protein expression of PKA, CREB and phosphorylated CREB in the hippocampus

**Figure 7:**
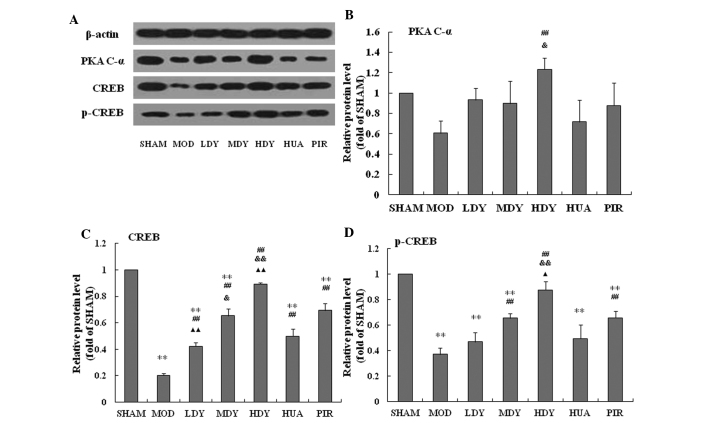
Protein expression of PKA, CREB and p-CREB in the hippocampus. (A) After treatment and subsequent memory evaluation, hippocampi were harvested and the protein expression was analyzed by western blotting. (B-D) Quantified protein levels of (B) PKA (C) CREB and (D) p-CREB normalized to β-actin. Values are expressed as the mean ± standard error. ^**^P<0.01 vs. SHAM; ^##^P<0.01 vs. MOD; ^&^P<0.05, ^&&^P<0.01 vs. HUA; ^▲^P<0.05, ^▲▲^P<0.01 vs. PIR. MOD, model group; LDY, low-dose YXJF group; MDY, medium-dose YXJF group; HDY, high-dose YXJF group; HUA, huperzine A group; PIR, piracetam group; YXJF, Yifei Xuanfei Jiangzhuo formula; PKA, protein kinase A; p-CREB, phosphorylated cyclic adenosine monophosphate-responsive element binding protein.

The present study further analyzed the protein expression of PKA, CREB and p-CREB in the hippocampus. Compared to the sham group, cerebral ischemia/reperfusion decreased the protein expression of the PKA C-α sub-unit (Fig. 7A and B) and CREB (Fig. 7A and C), and reduced CREB phosphorylation (Fig. 7A and D). Of note, YXJF treatment significantly ameliorated PKA C-α sub-unit and CREB protein expression, and promoted CREB phosphorylation in a dose-dependent manner. Huperzine A significantly increased CREB protein expression, but did not obviously promote PKA C-α protein expression or CREB phosphorylation, while piracetam markedly enhanced CREB protein expression and its phosphorylation, and, to a certain extent, promoted PKA C-α protein expression. These results suggested that YXJF improved memory impairment of rats with cerebral ischemia/reperfusion, at least in part through enhancing PKA/CREB signal transduction.

## Discussion

Medications currently used in the treatment of VaD, including cholinesterase inhibitors such as donepezil and non-cholinergics such as memantine (26,27), have been proven to be efficient to a certain extent. Owing to the limitations of these drugs (28), however, an increasing number of patients and clinician have resorted to using herbal medications to treat VaD.

Chinese herbs have been widely used to treat VaD in clinical practice in China. Although a previous clinical study by our group showed that the Chinese herbal decoction YXJF was efficient in the treatment of VaD (18), further studies on YXJF are required to provide additional convincing evidence and reveal the underlying mechanism of action. It is well known that cerebral hypoperfusion is a common physiopathological condition contributing to neurodegenerative diseases including VaD (29). Therefore, a rat model of VaD was successfully induced in the present study by repeated occlusion of the common carotid artery followed by reperfusion, which resulted in memory impairment, which was in accordance with the results of previous studies (20,21). Administration of YXJF significantly alleviated memory impairment in the model rats to a similar extent to that of the treatment with huperzine A and piracetam, the two currently used anti-dementia drugs (30,31), in agreement with the previous study by our group (18). These results suggested that YXJF has marked anti-VaD activity and is an alternative choice for the treatment of VaD.

The hippocampus, the key site of memory function, is highly sensitive to ischemia/hypoxia, and usually suffers the greatest injury during cerebral hypoperfusion (28,32). Apoptosis is an essential function for the normal development of any multicellular organism. In healthy organisms, apoptotic and anti-apoptotic proteins are balanced; however, through shifting this equilibrium, apoptosis can be upregulated during degenerative conditions and amplification of apoptosis through factors including pathological stimuli contributes to autoimmune disorders and cognitive deficits (33–35). It is generally accepted that cerebral ischemia/reperfusion injury is closely associated with apoptosis, thus resulting in the death of hippocampal CA1 neurons (36). To reveal the mechanism of action of YXJF, the present study assessed the occurrence of apoptosis in the hippocampal CA1 region through investigating the neuronal ultrastructure by TEM as well as TUNEL staining. As expected, cerebral ischemia/reperfusion caused apoptosis in the hippocampal CA1 region, and YXJF inhibited apoptosis in a dose-dependent manner. The present study used huperzine A and piracetam as positive controls for testing the efficacy of YXJF. Huperzine A is an inhibitor of acetylcholinesterase (AChE) isolated from Chinese folk medicine *Huperzia serrata* and piracetam is a derivative of the neurotransmitter gamma-aminobutyric acid (GABA); the two drugs have been proved to perform anti-apoptotic functions (37,38) and are widely applied in the clinical treatment of brain dysfunction. The positive control drugs indeed restrained apoptosis to a similar extent to that of YXJF, suggesting the anti-apoptotic action of YXJF.

It is well known that apoptosis is a strictly controlled process involving multiple changes in the expression of certain proteins, including Bcl-2 family proteins. Bcl-2 and Bax belong to the Bcl-2 family, and Bcl-2 exerts a survival function in response to a wide range of apoptotic stimuli through inhibition of Bax translocation and mitochondrial cytochrome *c* release (39), while Bax forms oligomers and translocates from the cytosol to the mitochondrial membrane upon apoptotic stimulation, thus leading to increases in mitochondrial membrane permeability, the release of cytochrome *c* from mitochondria, and subsequent activation of the apoptotic program (40,41). In addition, c-Jun, a member of the Jun family, dimerizes with Fos to form activator protein-1, a transcription factor that regulates genes and subsequently exerts diverse biological functions, including cell proliferation, differentiation and apoptosis (42). To further reveal the mechanism by which YXJF inhibits apoptosis, the expression of Bax, c-Jun and Bcl-2 was determined by western blot anaysis. In the present study, cerebral ischemia/reperfusion caused an increase in the protein expression of Bax and c-Jun, as well as a reduction in Bcl-2 expression in the hippocampus. Previous studies reported that upregulating the expression of Bcl-2 can protect neuronal cells from apoptosis (43), and that decreased Bax or increased Bcl-2 indicate anti-apoptotic neuroprotective effects in a rat model of VaD (44), while the activity of c-Jun was found to be increased in rats with cerebral ischemia/reperfusion (45), which was in accordance with the results of the present study. Of note, administration of YXJF decreased the protein expression of Bax and c-Jun, and increased Bcl-2 expression in the hippocampus. The results of the present study implied that YXJF alleviates memory impairment, at least in part, through inhibiting apoptosis in rats with cerebral ischemia reperfusion.

In the present study, huperzine A and piracetam were used as positive control drugs for YXJF; the former is an inhibitor of AChE, while the latter is a derivative of GABA. The improvement of memory impairment and apoptosis by huperzine A and piracetam clearly confirmed the potency of the decoction. According to previous studies, huperzine A was able to inhibit the upregulation of Bax and c-Jun, and reverse the downregulation of Bcl-2 *in vivo* and *in vitro* (37,46), while piracetam attenuates increases in the expression of Bax (47). In the present study, however, piracetam markedly inhibited Bax and c-Jun protein expression in hippocampi of rats with cerebral ischemia/reperfusion, and significantly increased Bcl-2, while huperzine A only had a marked effect in terms of decreasing Bax levels. Due to the similar effects on the protein expression of Bax, c-Jun and Bcl-2 between YXJF and piracetam, it was inferred that the two drugs may share a similar mechanism of action of alleviating memory impairment in rats following cerebral ischemia/reperfusion.

It is generally accepted that hippocampal cAMP/PKA/CREB signaling has a key role in memory formation (16,17). In this pathway, PKA is composed of two regulatory sub-units (R) and two catalytic sub-units (C). The C sub-unit exists in three isoforms (C-α, C-β, and C-γ). In the inactive state, the R sub-units block the active sites on the C sub-units. Upon increases in cAMP levels, the binding between cAMP and R sub-units reduces the auto-inhibitory contact, and active monomeric C sub-units are released, thus leading to the activation of PKA. Activated PKA promotes CREB phosphorylation at Ser133 (48) and subsequently regulates memory formation. Nefiracetam, a piracetam-like drug, was reported to improve memory function via cAMP/PKA/CREB signaling in rats with sustained cerebral ischaemia, showing increased cAMP levels, PKA C-α/β protein expression and CREB phosphorylation (17). Moreover, piracetam increased the cAMP concentration in the brain of guinea pigs (49), and promoted PKA C-β and CREB phosphorylation in aged rats (50). Therefore, piracetam improves memory impairment, partly through enhancing cAMP/PKA/CREB signaling. In the present study, piracetam was shown to have these effects, and YXJF treatment similarly increased PKA C-α and CREB protein expression, and promoted CREB phosphorylation. The results of the present study further confirmed that YXJF shares similar mechanisms of action with piracetam regarding their improvement of memory impairment in rats following cerebral ischemia/reperfusion.

In conclusion, the present study indicated that the Chinese herbal decoction YXJF improves memory impairment through anti-apoptotic mechanisms and through enhancing PKA/CREB signal transduction in rats with cerebral ischemia/reperfusion, which suggested the potential uses of YXJF, or compounds derived thereof, against cognitive deficits in VaD.
